# Oxidation of caspase-8 by hypothiocyanous acid enables TNF-mediated necroptosis

**DOI:** 10.1016/j.jbc.2023.104792

**Published:** 2023-05-06

**Authors:** Stephanie M. Bozonet, Nicholas J. Magon, Abigail J. Schwartfeger, Andreas Konigstorfer, Sarah G. Heath, Margreet C.M. Vissers, Vanessa K. Morris, Christoph Göbl, James M. Murphy, Guy S. Salvesen, Mark B. Hampton

**Affiliations:** 1 Mātai Hāora - Centre for Redox Biology and Medicine, Department of Pathology and Biomedical Science, University of Otago, Christchurch, New Zealand; 2 School of Biological Sciences, University of Canterbury, Christchurch, New Zealand; 3 Inflammation Division, The Walter and Eliza Hall Institute of Medical Research, Parkville, Victoria, Australia; 4 Department of Medical Biology, University of Melbourne, Parkville, Victoria, Australia; 5 Sanford Burnham Prebys Medical Discovery Institute, La Jolla, California, USA

**Keywords:** cell death, redox signaling, hypothiocyanous acid, thiol oxidation, inflammation

## Abstract

Necroptosis is a form of regulated cell death triggered by various host and pathogen-derived molecules during infection and inflammation. The essential step leading to necroptosis is phosphorylation of the mixed lineage kinase domain-like protein by receptor-interacting protein kinase 3. Caspase-8 cleaves receptor-interacting protein kinases to block necroptosis, so synthetic caspase inhibitors are required to study this process in experimental models. However, it is unclear how caspase-8 activity is regulated in a physiological setting. The active site cysteine of caspases is sensitive to oxidative inactivation, so we hypothesized that oxidants generated at sites of inflammation can inhibit caspase-8 and promote necroptosis. Here, we discovered that hypothiocyanous acid (HOSCN), an oxidant generated *in vivo* by heme peroxidases including myeloperoxidase and lactoperoxidase, is a potent caspase-8 inhibitor. We found HOSCN was able to promote necroptosis in mouse fibroblasts treated with tumor necrosis factor. We also demonstrate purified caspase-8 was inactivated by low concentrations of HOSCN, with the predominant product being a disulfide-linked dimer between Cys360 and Cys409 of the large and small catalytic subunits. We show oxidation still occurred in the presence of reducing agents, and reduction of the dimer was slow, consistent with HOSCN being a powerful physiological caspase inhibitor. While the initial oxidation product is a dimer, further modification also occurred in cells treated with HOSCN, leading to higher molecular weight caspase-8 species. Taken together, these findings indicate major disruption of caspase-8 function and suggest a novel mechanism for the promotion of necroptosis at sites of inflammation.

Multicellular organisms have tightly controlled processes for the removal of damaged or unwanted cells. Apoptosis is the best characterized cell death program and is associated with activation of proteolytic caspases and packaging of the cell for clearance. Necrosis, in contrast, involves cell swelling, loss of membrane integrity, and the release of intracellular contents. A regulated form of necrosis, termed necroptosis (1), can be triggered by death receptor ligands and various bacterial and viral constituents *via* receptor-interacting protein kinase 3 (RIPK3)-mediated phosphorylation of the mixed lineage kinase domain-like (MLKL) pseudokinase (2, 3). Phosphorylated MLKL forms oligomers that associate with membranes and mediate cell death (4, 5, 6, 7).

Caspase-8 promotes death receptor-mediated apoptosis (8) and also blocks necroptosis by binding and cleaving signaling proteins that include components of the RIPK1/RIPK3/MLKL necrosome complex (9, 10, 11, 12, 13). In experimental models, broad spectrum caspase inhibitors are included to enable necroptosis, but less is known about the regulation of caspase activity under physiological conditions. Caspases have a nucleophilic cysteine residue at their active site, and *in vitro* activity assays require addition of reductants to prevent oxidative inactivation (14). Our previous demonstrations of oxidant sensitivity (15, 16, 17) led us to hypothesize that exposure of cells to death receptor ligands in the presence of oxidative stress inactivates caspase-8 and thereby promotes the induction of necroptosis.

At sites of inflammation, activated neutrophils are a major source of oxidants, which they use to attack pathogenic microbes (18). Upon activation, their NOX2 complex assembles and converts oxygen to superoxide, which dismutates to hydrogen peroxide (H_2_O_2_) and can then be converted to hypochlorous acid (HOCl) and hypothiocyanous acid (HOSCN) by the heme enzyme myeloperoxidase (18). HOSCN is also produced by eosinophil peroxidase and by lactoperoxidase (LPO) in mucosal fluids. It specifically targets sulfhydryl groups and is therefore of particular interest in the context of caspase-8 (19). HOSCN has previously been shown to prevent caspase-3 activation and inhibit existing caspase-3 activity in endothelial cells when added before or after apoptosis initiated by growth factor removal (17).

In this study, we show that sublethal doses of HOSCN are able to mimic a synthetic caspase inhibitor in switching tumor necrosis factor (TNF)-mediated apoptosis to necroptotic cell death, and we identify the specific modification of caspase-8 that is responsible for loss of enzyme activity. We propose that oxidants produced at inflammatory sites by activated neutrophils, particularly HOSCN, act as physiological modulators of cell death pathways through the ability to inactivate caspase-8. The resultant promotion of necroptosis will have significant impacts for the resolution of inflammation.

## Results

### Inhibition of caspase activity and induction of necroptosis by HOSCN in fibroblasts co-incubated with TNF

**Figure 1:**
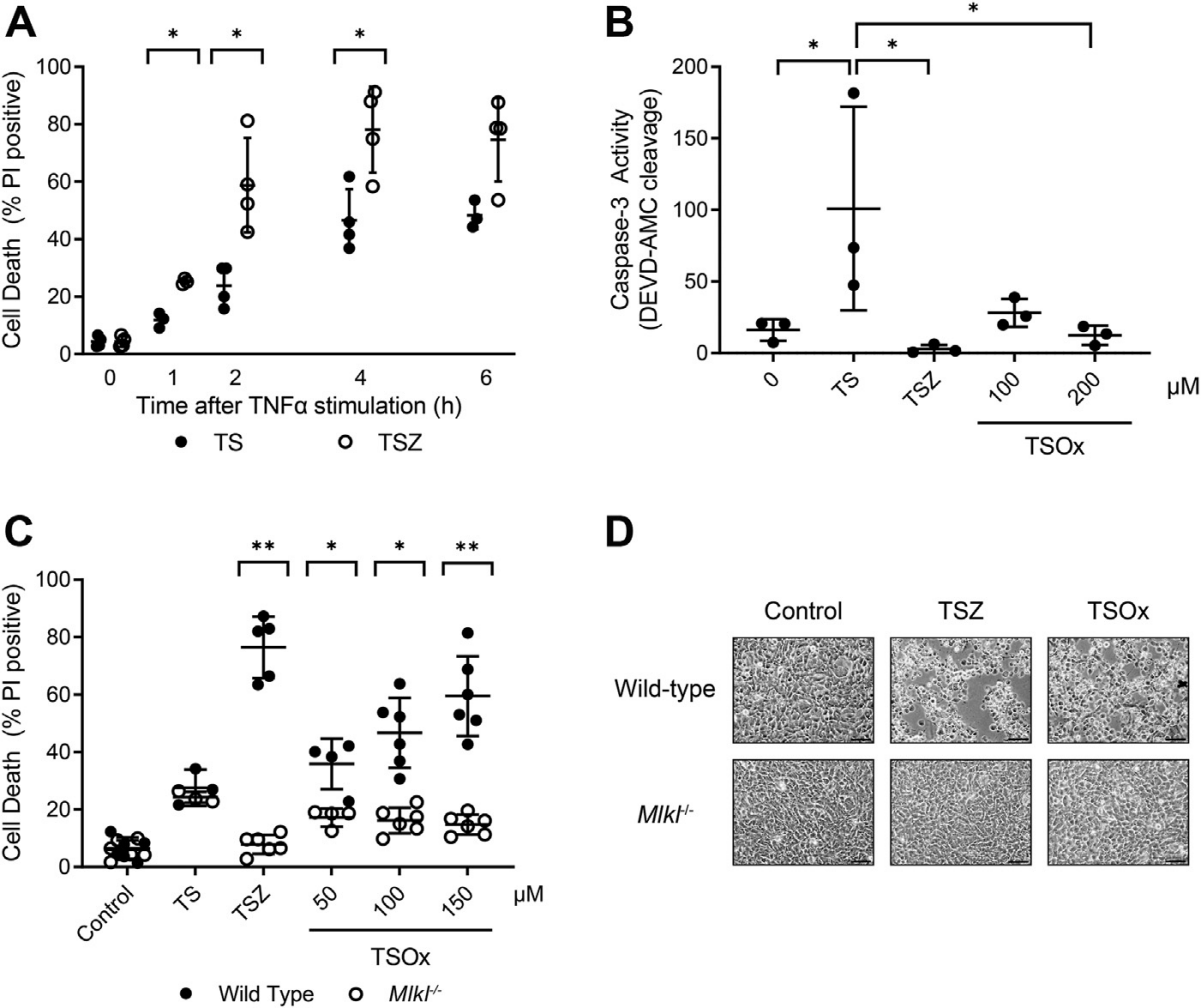
**Effect of HOSCN on TNF-mediated cell death.** (*A)* MDF incubated with apoptotic (TS) or necroptotic (TSZ) stimuli for up to 6 h before cell death was measured by propidium iodide uptake in combination with flow cytometry. Data are from four independent experiments, and *asterisks* show statistically significant differences between TS and TSZ (∗*p* < 0.05, multiple paired *t* tests). Cells were also incubated for 3 h with TS, TSZ, or TSOx to investigate the impact of the caspase inhibitor zVAD-fmk (Z, 20 μM) or the oxidant HOSCN (Ox) on TS-induced caspase activation and cell death. (*B)* caspase-3-dependent DEVDase activity was measured. Data are from three independent experiments; *asterisks* indicate treatments are significantly different (∗*p* < 0.05) from TS-treated cells (one-way repeated measure ANOVA with Dunnett’s multiple comparison test). (*C)* wildtype and *Mlkl*^*−/−*^ cell death was measured. Data are from 3 to 6 independent experiments. Significant differences were observed between wildtype and *Mlkl*^*−/−*^ cells (∗*p* < 0.05, ∗∗*p* < 0.001, multiple paired *t* tests). (*D)* representative images of cell morphology after 3 h incubation with (HOSCN, 150 μM), 10× objective, scale bar = 200 μm. HOSCN, hypothiocyanous acid; MDF, mouse dermal fibroblast; TS, TNF and Smac mimetics.

The combined addition of TNF and second mitochondrial-derived activator of caspase (Smac) mimetic (TS) to mouse dermal fibroblasts (MDFs) resulted in cell death that was accelerated with the addition of the caspase inhibitor zVAD-fmk (Z) (Fig. 1*A*). Incubation with TS resulted in detectable caspase-3 activity (Fig. 1*B*) and death of 25% of the cells within 3 h (Fig. 1*C*), whereas inclusion of Z completely blocked caspase activation (Fig. 1*B*), yet 75% of the cells still died within 3 h (Fig. 1*C*). MDF cells derived from MLKL knockout mice were protected from TSZ-mediated cell death, confirming that the rapid cell death in this model was due to necroptosis (Fig. 1, *C* and *D*).

To determine if HOSCN could replace the caspase inhibitor in switching apoptosis to necroptosis, cells were treated with various concentrations of oxidant for 10 min prior to the addition of TNF (TSOx). Inhibition of TS-mediated caspase activation was observed with 100 μM HOSCN (Fig. 1*B*), and while not as effective as the synthetic caspase inhibitor, this corresponded to increased cell death of wildtype MDF but not *Mlkl*^*−/−*^ cells (Fig. 1*C*). HOSCN is known to be cytotoxic by itself at high doses, resulting in the rounding and detachment of adherent cells (17). The doses of HOSCN that inhibited caspase activity and protected *Mlkl*^*−/−*^ cells from apoptosis did not cause cell death on their own and had no obvious morphological effect on the cells (Fig. 1*D*).

**Figure 2:**
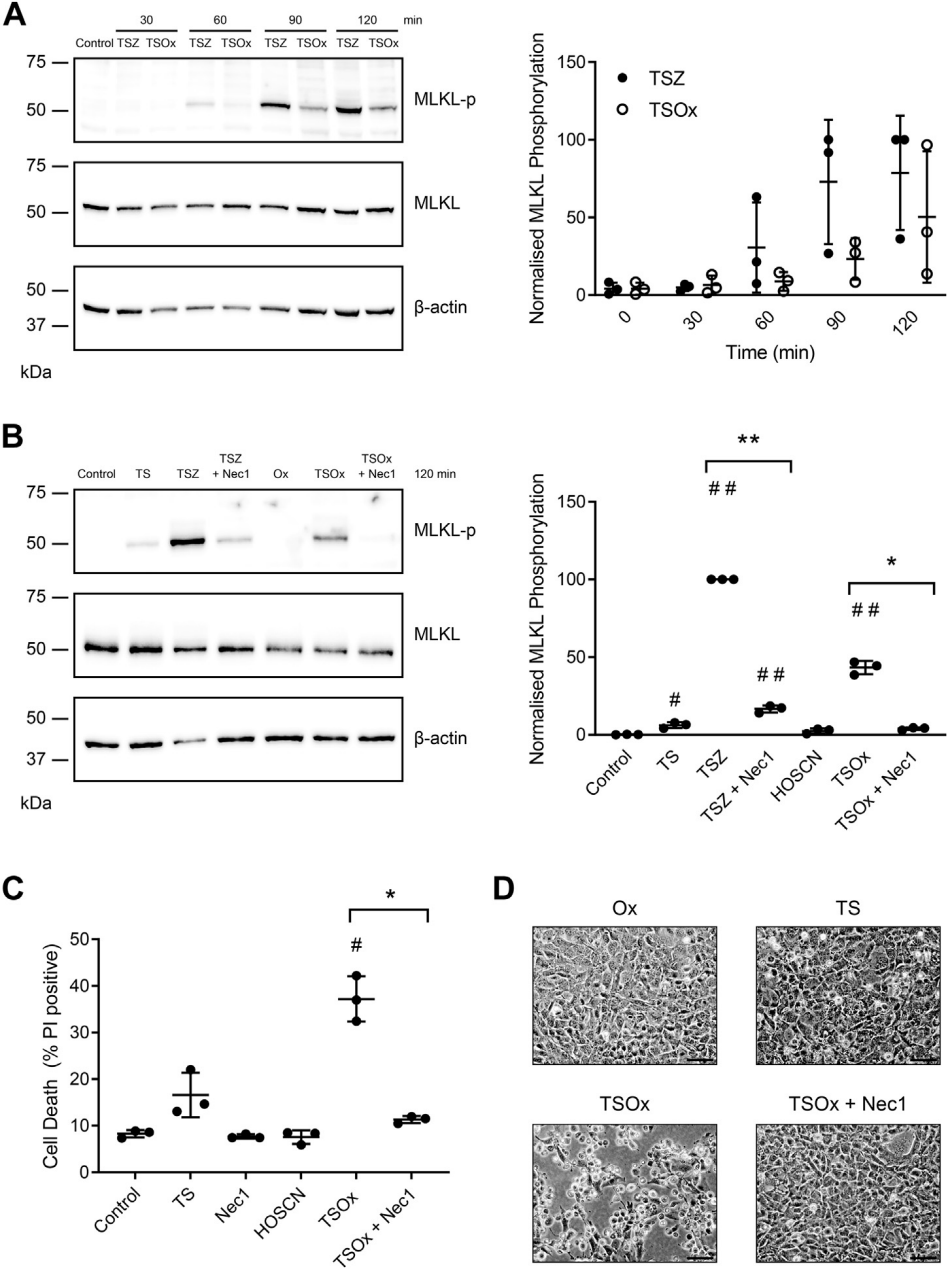
**MLKL phosphorylation and necroptotic morphology in mouse cells exposed to TSOx.** (*A)* MDFs were incubated with either TSZ or TSOx (HOSCN, 150 μM) for the indicated times, then lysed, and immunoblotted for MLKL (phosphorylated and total) and β-actin (loading control). The graphical representation of MLKL phosphorylation, adjusted for the total amount of MLKL, was measured by densitometry and presented as “% of control.” Despite a trend, results were not statistically significant (two-way ANOVA with Dunnett’s multiple comparison). (*B)* MDFs were incubated for 120 min with apoptotic induction (TS) or TSOx with and without the necroptosis inhibitor Nec1. Cell lysates were immunoblotted and measured by densitometry as above. A significant difference was observed with Nec1 (two-tailed, paired *t* test, ∗*p* < 0.05 and ∗∗*p* < 0.01), and # indicates a significant difference from control (two-way ANOVA with Dunnett’s multiple comparison #*p* < 0.05, ##*p* < 0.0001). (*C)* cells were incubated for 3 h under the indicated conditions, then harvested, and analyzed for cell death by flow cytometry with PI. # indicates a significant difference (*p* < 0.005) from control (one-way ANOVA with Dunnett’s multiple comparison test), ∗two-tailed paired *t* test shows a significant difference (*p* < 0.05) between TSOx and TSOx+Nec1. All data are from three independent experiments. (*D)* representative images of cells from *C* captured using the 10× objective, scale bar = 200 μM. HOSCN, hypothiocyanous acid; MDF, mouse dermal fibroblast; MLKL, mixed lineage kinase domain-like; Smac, second mitochondrial-derived activator of caspase; TNF, tumor necrosis factor; TS, TNF and Smac mimetics; PI, propidium iodide.

Phosphorylation of MLKL by RIPK3 is essential for necroptosis (2, 20). Western blotting with antibodies specific to the phosphorylated form of MLKL revealed a time-dependent increase in MLKL phosphorylation in cells exposed to either TSZ or TSOx (Fig. 2*A*). This was prevented by Nec1, an inhibitor of RIPK1, the apical kinase in the necroptosis pathway, confirming that TSOx-mediated MLKL phosphorylation occurs by the canonical RIPK1–RIPK3 cascade (Fig. 2*B*). Consistent with blockade of RIPK1–RIPK3, Nec1 was able to block TSOx-mediated cell death (Fig. 2*C*).

### Inhibition of recombinant caspases by HOSCN

**Figure 3:**
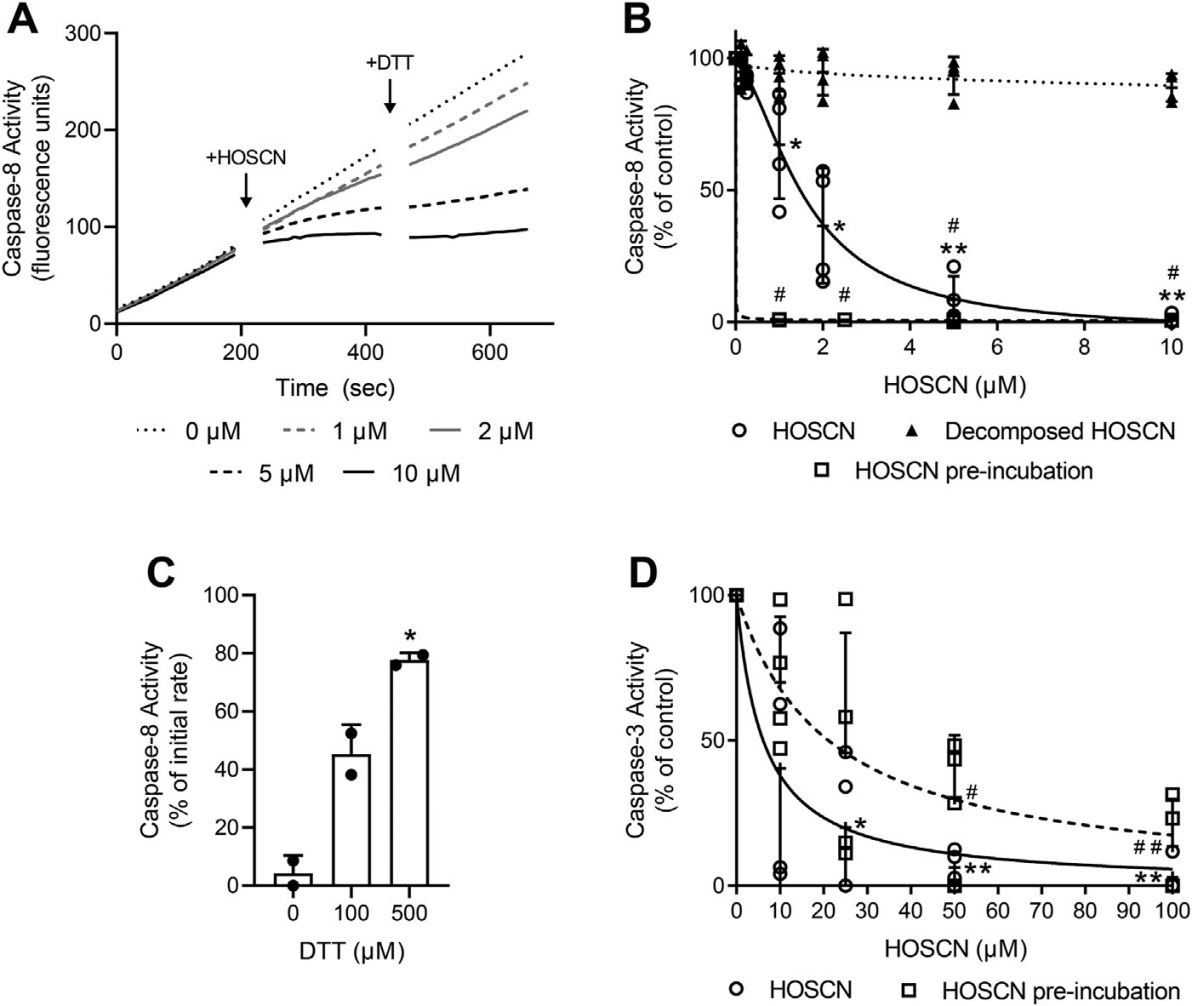
**Inhibition of recombinant caspases by HOSCN.** (*A)* representative trace of recombinant caspase-8 activity showing accumulation of the fluorescent cleavage product: enzyme was added to assay buffer containing substrate, and the reaction was monitored before addition of HOSCN (0–10 μM), then DTT (100 μM). (*B)* the reaction was monitored for 3 min, then HOSCN (fresh or decomposed) was added, and the reaction monitored for a further 3 min. Enzyme activity was calculated for the “HOSCN” and “decomposed HOSCN” phases and each expressed as a percentage of activity of the untreated enzyme (“0” buffer only). The enzyme was also preincubated with HOSCN for 3 min before addition of substrate (“HOSCN pre-incubation”). Analysis (one-way ANOVA with Dunnett’s multiple comparison test) shows a significant difference from the control for HOSCN; ∗*p* < 0.05 and ∗∗*p* < 0.001; and “HOSCN pre-incubation”, # *p* < 0.01. Data are from 4 independent experiments. (*C)* to test the ability of DTT to restore activity, the reaction was measured (for 3 min) 30 min after the addition of DTT (100 or 500 μM). Activity is presented as a % of the activity rate prior to HOSCN exposure: ∗*p* < 0.05 indicates a significant difference from “0 DTT” (one-way ANOVA with Dunnett’s multiple comparison test). Data are from two independent experiments. (*D)* recombinant caspase-3 was added to assay buffer containing substrate, the reaction was monitored for 3 min before HOSCN was added, and the reaction was monitored for a further 3 min. The enzyme was also preincubated with HOSCN for 10 min before addition of the substrate (“preincubated”) and the reaction similarly monitored. Enzyme activity was calculated for the “HOSCN” phases and each expressed as a percentage of the activity of control (“0” buffer only). A statistically significant difference from “0” is shown for HOSCN (∗*p* < 0.05, ∗∗*p* < 0.001) and for “HOSCN pre-incubation" # *p* < 0.05, ## *p* < 0.001 (one-way ANOVA with Dunnett’s multiple comparison test). Data are from 3 to 4 independent experiments. HOSCN, hypothiocyanous acid; DTT, dithiothreitol.

Since our cellular studies are consistent with HOSCN influencing cell death programs by targeting caspase-8, we proceeded to examine the capacity of HOSCN to directly inhibit recombinant caspase-8. Enzyme activity was monitored in the presence of an artificial substrate as illustrated in Figure 3*A*. The caspase buffer included 1 μM dithiothreitol (DTT) to prevent inactivation of caspase-8 by trace oxidants in the buffers. Despite the presence of DTT, inhibition was observed with 1 to 2 μM HOSCN, and complete inhibition occurred upon addition of 10 μM HOSCN (Fig. 3, *A* and *B*). The inhibitory effect of HOSCN was even more potent if the enzyme was treated with HOSCN prior to the addition of substrate (Fig. 3*B*). In aqueous solutions, HOSCN degrades to a range of chemical species (19). Inhibitory activity was lost if HOSCN was left for 5 h prior to use, indicating that products of HOSCN decomposition had no effect on the enzyme. Caspase-8 activity was not immediately restored by the addition of DTT (Fig. 3*A*), but longer incubation with higher concentrations of DTT was able to restore activity (Fig. 3*C*).

Inhibition of recombinant caspase-3 by HOSCN was also assessed. Unlike caspase-8, a spin column could be used to remove DTT from the enzyme preparation immediately prior to use without major loss of activity. Caspase-3 was inhibited by HOSCN at low micromolar concentrations (Fig. 3*D*), but the enzyme was markedly less sensitive than caspase-8. When preincubated with HOSCN in the absence of substrate, caspase-3 was less sensitive to inhibition (Fig. 3*D*), consistent with published findings that the presence of substrate sensitizes caspase-3 to oxidative inactivation (16).

### Characterizing caspase modification by HOSCN

**Figure 4:**
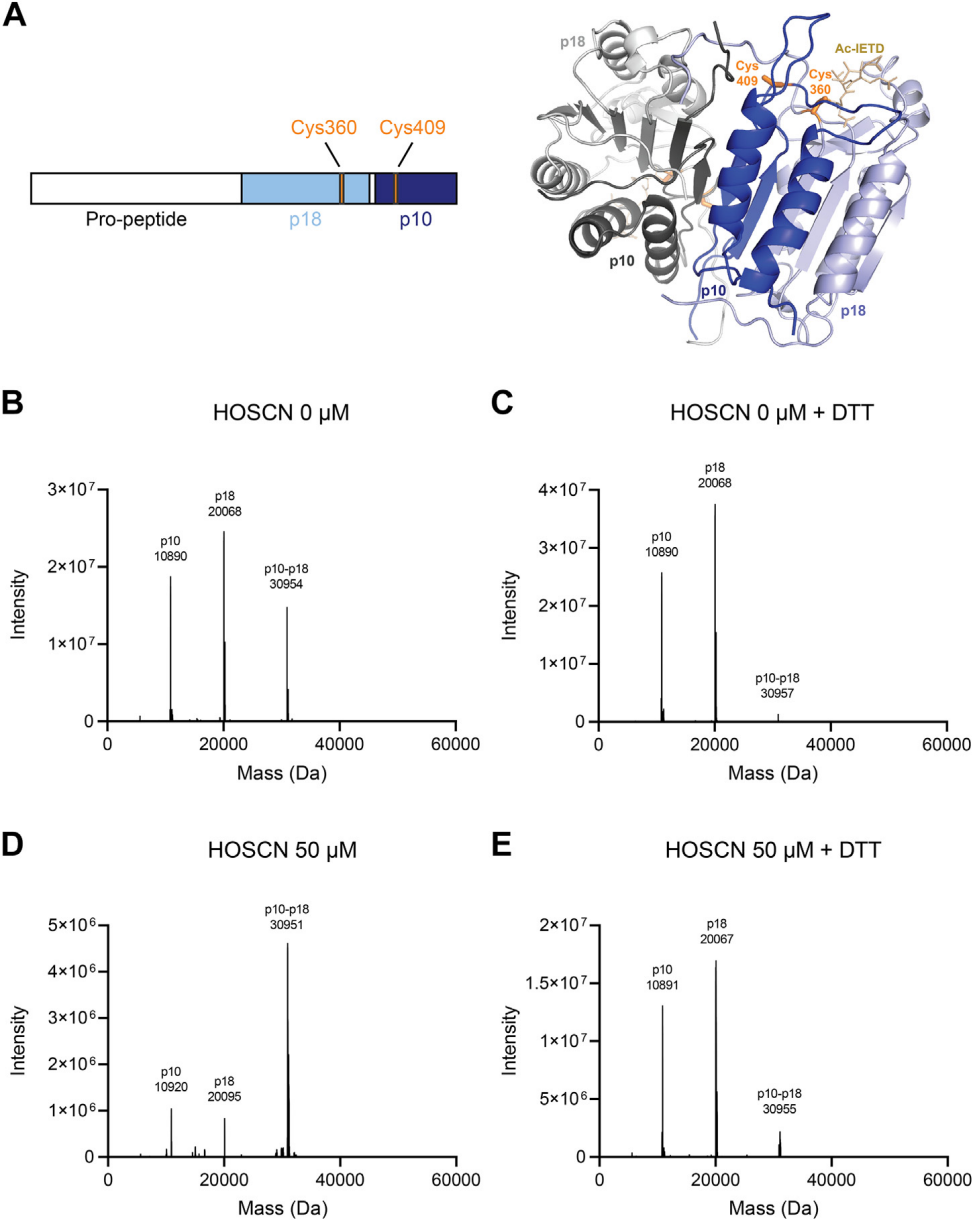
**Molecular mass of caspase-8 in the presence of HOSCN.** (*A)* schematic structure of caspase-8 showing the propeptides (1–216, 375–384 in *white*), the large subunit, p18, (217–374 in *light blue*), and the small subunit, p10, (385–479 in *dark blue*). The crystal structure is from PDB: 1QTN (43). (*B*–*E)* recombinant caspase-8 was treated with HOSCN [0 μM (*B* and *C*) or 50 μM (*D* and *E*)] and incubated at room temperature for 10 min. Samples were split and half of each exposed to DTT (2.5 mM) (*C* and *E*) for 30 min at room temperature. Samples were analyzed by intact protein LC/MS. Spectra recorded over the full width of the protein peak were averaged and deconvoluted. Deconvoluted spectra are representative of at least three separate experiments. The expected molecular weights of the different species are in Table S2. HOSCN, hypothiocyanous acid; DTT, dithiothreitol.

We predicted the active site cysteine (Cys360) in the large subunit (p18) of caspase-8 to be the primary target of HOSCN. A crystal structure of caspase-8 indicates a neighboring Cys409 that could form a disulfide bond with Cys360 (Fig. 4*A*). Since this is on the small subunit (p10) of caspase-8, oxidation would result in an intermolecular disulfide.

Intact protein LC/MS was used to analyze the modification of caspase-8 by HOSCN. A representative deconvoluted mass spectrum is shown for untreated caspase-8 without (Fig. 4*B*) and with DTT (Fig. 4*C*). Untreated caspase-8 comprises three major species corresponding to the small (p10) and large (p18) subunits of caspase-8, as well as a higher molecular weight species (∼31 kDa) whose abundance was markedly decreased in the presence of DTT. Treatment with HOSCN increased the abundance of the higher molecular weight species (Fig. 4*D*), and this was reversed by DTT (Fig. 4*E*), consistent with the formation of an intersubunit disulfide. The deconvoluted mass of the high molecular weight species (30,951 Da) is close to the theoretical value of a disulfide-linked p10:p18 heterodimer (30,953 Da). In addition, the small and large subunit peaks increased in mass following HOSCN treatment, consistent with formation of sulfinic acids. Digestion with trypsin and analysis by LC/MS revealed that the peak area of the p18 peptide containing sulfinic acid (VFFIQAC_360_(sulfinic acid)QGDNYQK) increased after treatment with HOSCN and was not altered by DTT (Fig. S2).

**Figure 5:**
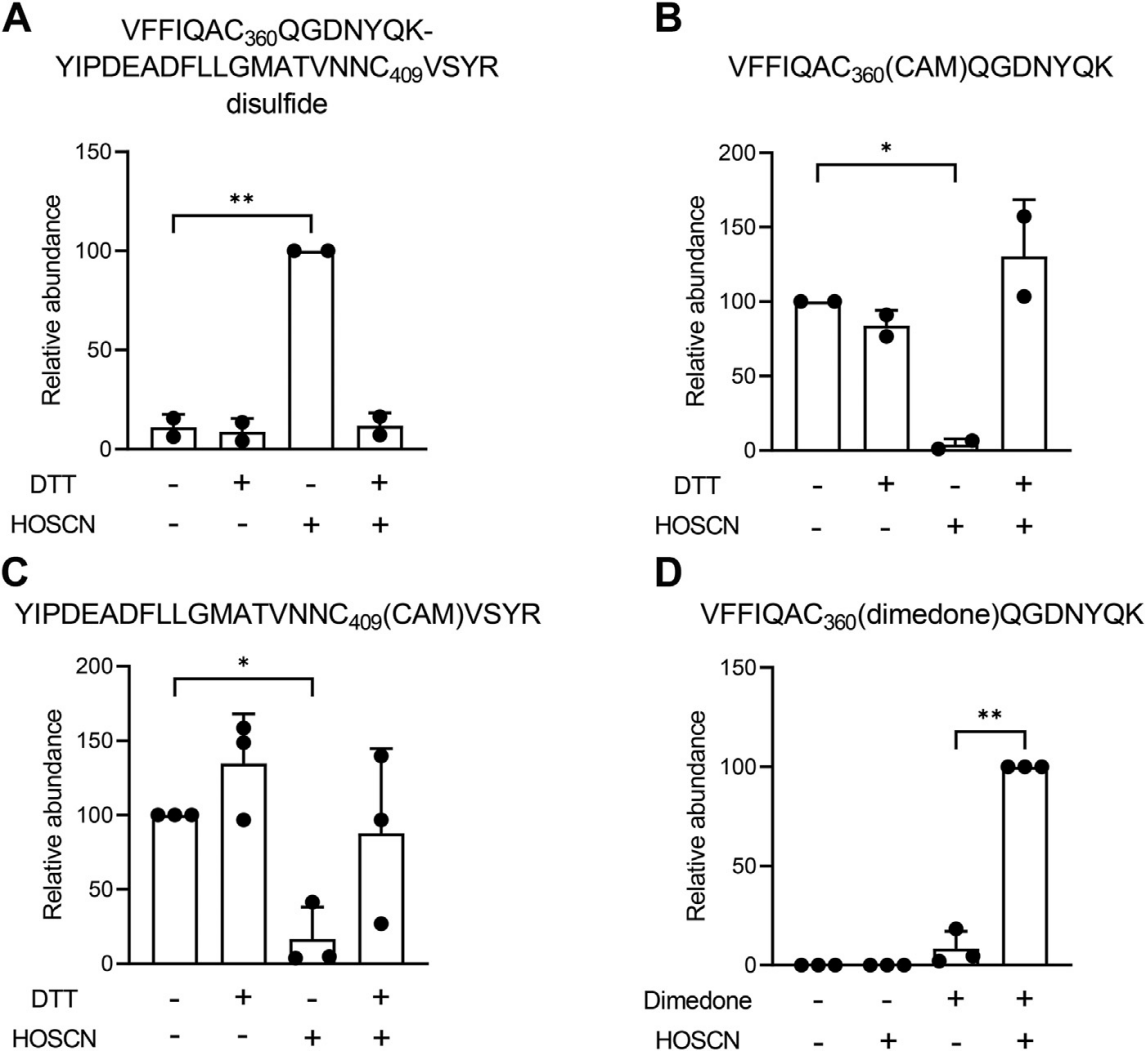
**Characterization of caspase-8 intermolecular disulfide resulting from exposure to HOSCN.***A*–*C*, recombinant caspase-8 was treated with HOSCN and then incubated with or without DTT. The protein was subsequently alkylated and digested with trypsin before analysis by LC-MS/MS. The relative peak areas for (*A*) the disulfide VFFIQAC_360_QGDNYQK-YIPDEADFLLGMATVNNC_409_VSYR and both peptides, (*B*) VFFIQAC_360_(CAM)QGDNYQK, and (*C*) YIPDEADFLLGMATVNNC_409_(CAM)VSYR were determined for each treatment. Significant differences from control were observed, ∗*p* < 0.05 and ∗∗*p* < 0.01 (two-way ANOVA with Dunnett’s multiple comparisons test). (*D)* recombinant caspase-8 was treated with HOSCN in the presence of dimedone, then alkylated and digested with trypsin, before analysis by LC-MS/MS. The peak area for the VFFIQAC_360_(dimedone)QGDNYQK peptide was determined for each treatment. A significant difference was observed between dimedone with and without HOSCN, ∗∗*p* = 0.003 (2-tailed, paired *t* test). CAM, carbamidomethylation resulting from treatment with iodoacetamide; DTT, dithiothreitol; HOSCN, hypothiocyanous acid.

To investigate the nature of the intermolecular disulfide, caspase-8 was incubated with HOSCN and digested with trypsin before analysis by LC/MS. Evidence was found for a disulfide bond between the active site Cys360 and Cys409. The peak area of the disulfide was found to be greatly increased after treatment with HOSCN and restored to control levels after the addition of DTT (Figs. S3 and 5*A*). The individual alkylated peptides, VFFIQAC_360_(carbamidomethyl)QGDNYQK and YIPDEADFLLGMATVNNC_409_(carbamidomethyl)VSYR were characterized, and complementary results were observed for their peak areas: their abundance was decreased upon treatment with HOSCN relative to the control and restored upon addition of DTT (Fig. 5, *B* and *C*). The fragmentation patterns for both peptides and the disulfide, as well as its MS^3^ spectrum, are shown in Fig. S3.

**Figure 6:**
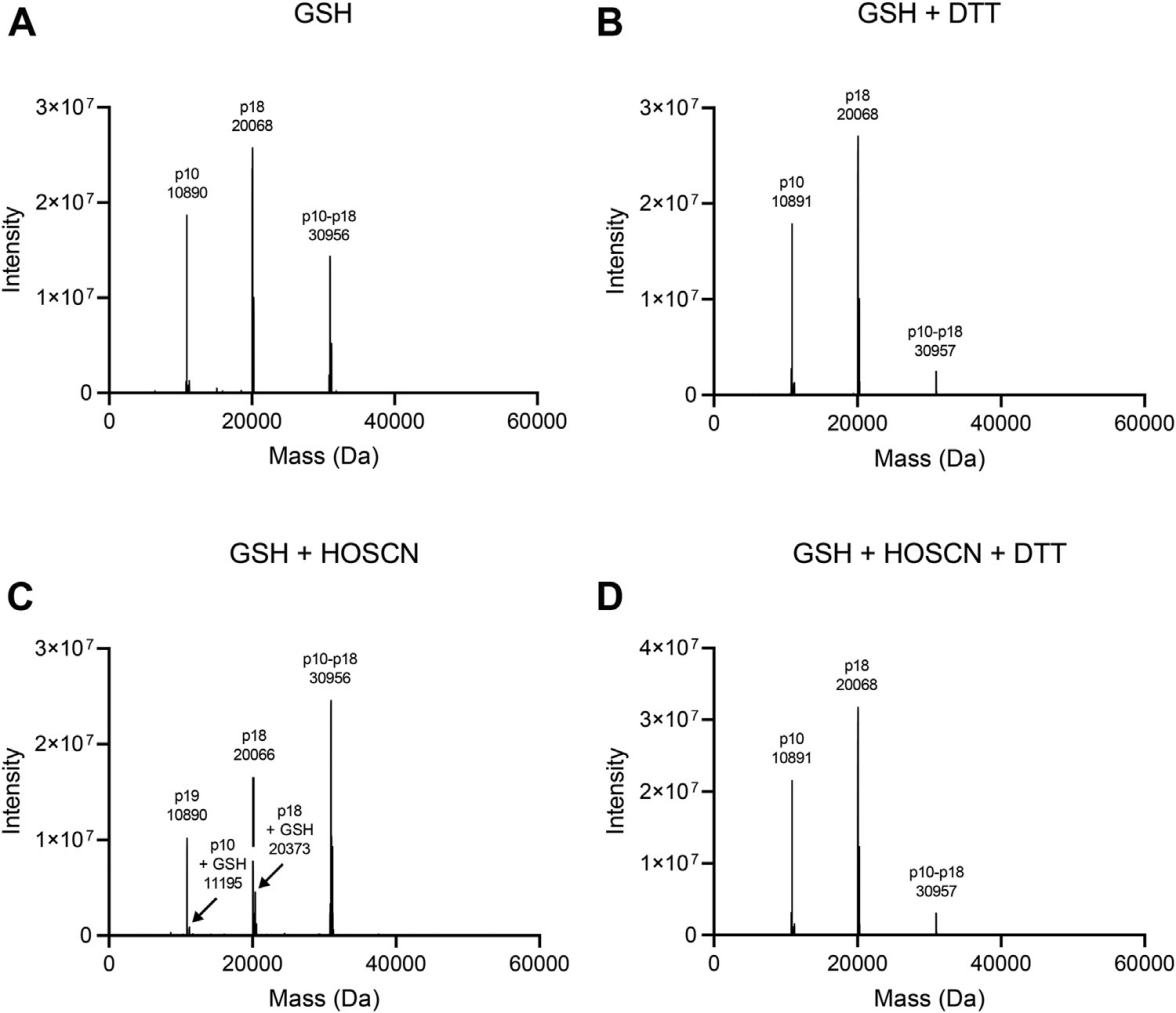
**Caspase-8 forms a mixed disulfide with GSH in the presence of HOSCN.***A*–*D*, recombinant caspase-8, in the presence of GSH (100 μM), was untreated (*A* and *B*) or treated with HOSCN (50 μM, *C* and *D*). Samples were split and half of each exposed to DTT (2.5 mM) (*B* and *D*) then analyzed by LC/MS. *Arrows* in (*C*) indicate addition of GSH to the small and large subunits of caspase-8. Spectra recorded over the full width of the protein peak were averaged and deconvoluted. Spectra are representative of two separate experiments. The expected molecular weights of the different species are in Table S2. GSH, glutathione; HOSCN, hypothiocyanous acid; DTT, dithiothreitol.

HOSCN reacts with cysteine to form a sulfenyl thiocyanate that can hydrolyze to a sulfenic acid (19). An adduct was formed on the active site cysteine when caspase-8 was treated with HOSCN (50 μM) in the presence of dimedone, a nucleophilic reagent that reacts with sulfenic acids (21) (Figs. 5*D* and S4). *In vitro* the sulfenic acid will react with Cys409 to form the intermolecular disulfide; however, under physiological conditions, the sulfenic acid could also react with reduced glutathione (GSH). Intact protein LC/MS was used to analyze the modification of caspase-8 by HOSCN in the presence of GSH. Shoulder peaks corresponding to the addition of GSH (indicated by arrows) were observed on both the small and large subunits after addition of HOSCN (Fig. 6*C*), with tryptic digests confirming that GSH formed a mixed disulfide with the active site cysteine of the large subunit (Fig. S5). However, the intermolecular disulfide remained the major product (Fig. 6*C*).

### HOSCN-induced formation of a caspase-8 intermolecular disulfide

**Figure 7:**
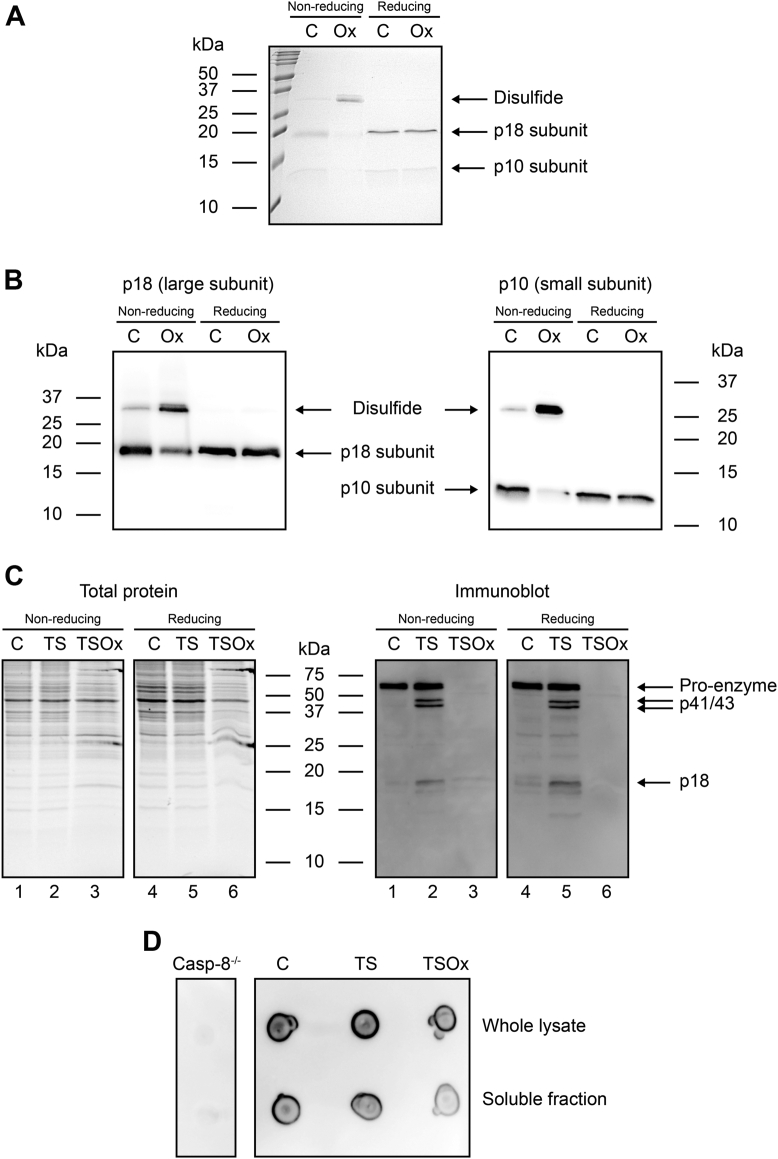
**Caspase-8 disulfide formation in the presence of HOSCN.***A* and *B*, recombinant caspase-8 was exposed to HOSCN (100 μM) for 10 min at room temperature, then separated by SDS-PAGE under non-reducing and reducing (+DTT) conditions, and (*A*) stained with Coomassie brilliant blue or (*B*) immunoblotted for caspase-8 (*left-hand panel*: p18, *right-hand panel*: p10). (*C)* Jurkat cells were incubated with TS for 2 h before the addition of HOSCN (TSOx, 150 μM) for 10 min, then lysed, and immunoblotted for caspase-8 (p18, p41/43, p55/57). Total proteins were visualized by UV imaging of trihalo-labeled tryptophan residues. (*D)* dot blot to confirm detection of caspase-8 by the same antibody as in C, with casp-8^−/−^ cell lysate as negative control. HOSCN, hypothiocyanous acid; DTT, dithiothreitol.

Our next goal was to seek evidence of caspase-8 oxidation in cells treated with HOSCN. First, the intermolecular disulfide between p10 and p18 subunits of recombinant protein was measured by non-reducing SDS-PAGE. Experiments were performed with the caspase assay buffer, which includes sucrose to stabilize the protein, as well as cell lysis buffer that would be necessary to detect caspase-8 in cells. Coomassie staining of recombinant caspase-8 showed the p10 and p18 subunits as well as a faint band at ∼30 kDa, representing the small proportion of the protein not reduced by the initial addition of DTT after thawing, and is consistent with that observed by LC/MS (Fig. 7*A*). After exposure to HOSCN, the intensity of the p10 and p18 bands was decreased and that of the higher molecular weight band (∼30 kDa) was increased. This was reversed by the addition of DTT, consistent with a disulfide bond linking the p10 and p18 subunits. Before extending the experiments to cells, we first confirmed that western blotting with caspase-8 antibodies could detect oxidized caspase-8 (Fig. 7*B*). The results mirrored what was observed with protein staining.

Oxidation of pro-caspase-8 in cells would generate an intramolecular disulfide that could not be detected with this methodology; therefore, Jurkat T lymphoma cells were incubated with TS for 2 h to induce caspase-8 cleavage (Fig. 7*C*, immunoblot lanes 2 and 5). Addition of HOSCN for 10 min resulted in a dramatic loss of both active and pro-caspase-8 subunits (Fig. 7*C*, immunoblot lanes 3 and 6), with neither form of the protein detectable after reduction with DTT (Fig. 7*C* immunoblot lane 6). Dot blotting was used to confirm that the antibody was able to bind to the HOSCN-treated caspase-8 (Fig. 7*D*). This indicates that treatment of cells with HOSCN results in more extensive modification to caspase-8 than just disulfide formation and is consistent with HOSCN-mediated modification of caspase-8 promoting necroptotic cell death.

**Figure 8:**
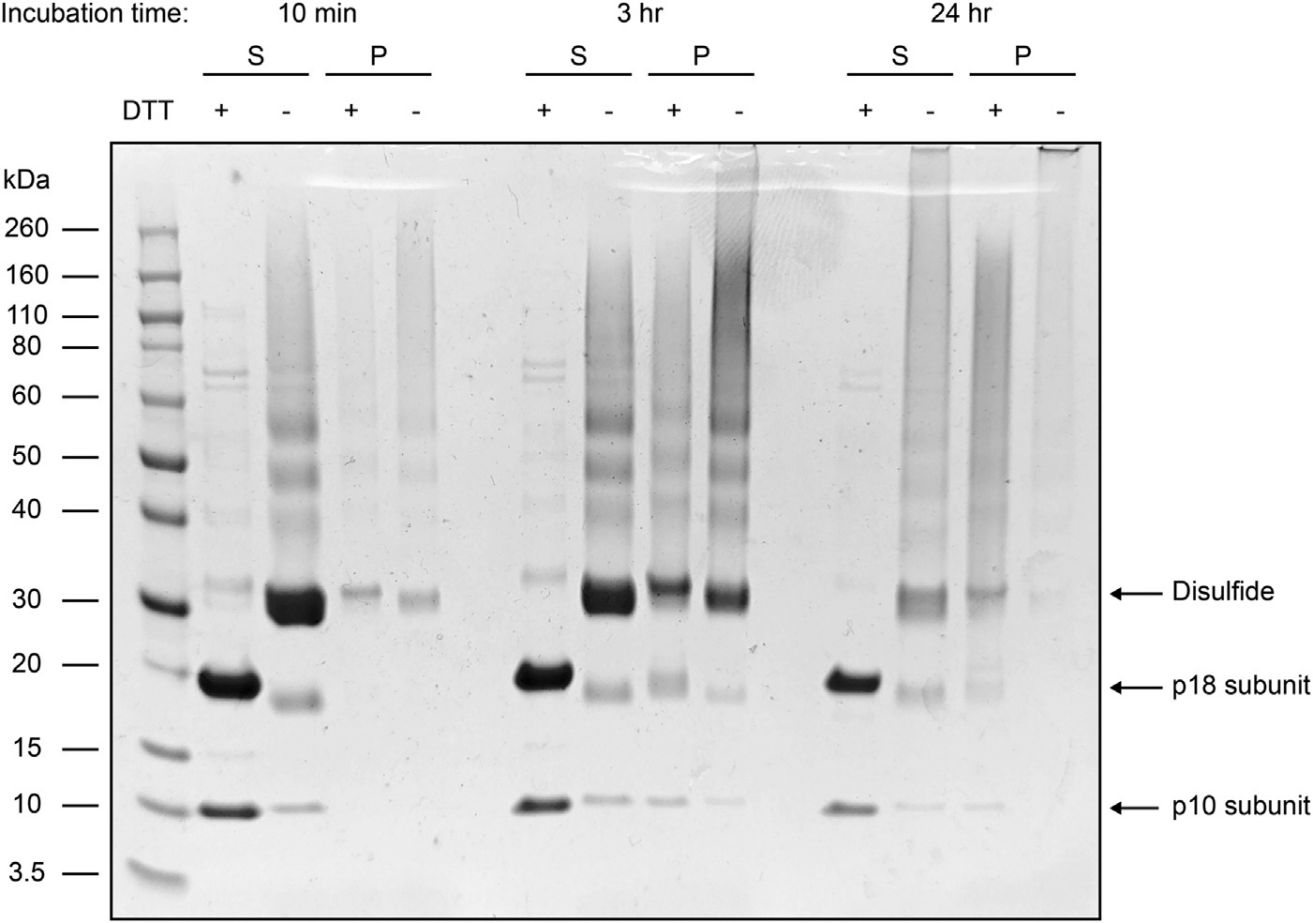
**Formation of high molecular weight species from HOSCN oxidation.** Recombinant caspase-8 was exposed to HOSCN (100 μM) and incubated for 10 min, 3 h, or 24 h before centrifugation at 21,000*g* for 1 h at 25 °C. The supernatant and pellet (S and P, respectively) were collected and applied to SDS-PAGE under reducing (+DTT) and non-reducing conditions and visualized using Coomassie brilliant blue staining. HOSCN, hypothiocyanous acid; DTT, dithiothreitol.

We hypothesized that the HOSCN-induced modification resulted in an increased molecular weight species that is unable to migrate into the polyacrylamide gel. To test this, we oxidized recombinant protein and analyzed it using gel filtration. This revealed that oxidation decreased the amount of monomeric protein, but no large species were observed, potentially because it was too large to enter the column (Fig. S6). Therefore, we oxidized recombinant caspase-8 protein for up to 24 h and centrifuged the samples to separate large aggregates. At longer time intervals, a visible pellet was observed, and both the supernatant and pellets were analyzed by SDS-PAGE, revealing that higher molecular weight species are formed over time (Fig. 8).

## Discussion

Necroptotic cell death results in cell rupture and the release of damage-associated molecular patterns, promoting an ongoing inflammatory response (22). This lytic form of cell death is thought to have evolved to assist with pathogen clearance, but excessive necroptosis is associated with the pathology of several diseases (23, 24, 25, 26). Caspase-8 is known to inactivate various necroptosis mediators (9, 10, 11, 12, 13). It has also been reported that caspase-8 blocks inflammasome activation (27), and in cells without the necessary necroptosis effectors, the removal of caspase-8 leads to pyroptosis (28, 29). In this study, we have shown that HOSCN, an oxidant generated by the innate immune system, is a potent inhibitor of caspase-8. When cells were exposed to HOSCN in the presence of a death receptor ligand, cell death was switched from apoptosis to a characteristic form of MLKL-dependent necroptosis. We therefore propose that HOSCN promotes inflammation through its ability to inhibit the various anti-inflammatory activities of caspase-8.

It has been known for some time that caspase inhibition leads to necroptosis (30, 31, 32). Caspase-8 knockout mice die during embryonic development from excessive necroptosis (9, 33), and human caspase-8 mutations are associated with immunodeficiency and inflammatory bowel disease (34, 35). Synthetic caspase inhibitors are required to enable necroptosis in experimental systems, but it is less clear how necroptosis is controlled under physiological conditions. A short isoform of the c-FLIP protein, a catalytically inactive homolog, blocks caspase-8 activation in cells (36). Viral caspase inhibitors have a similar effect, and more recently, phosphorylation of caspase-8 at Thr265 has been reported to inhibit caspase-8 activity and promote necroptosis (37). The reversible oxidation of specific cysteine residues provides an effective means for posttranslational modification of many different proteins, with oxidation leading to structural or enzymatic effects in the target proteins. Studies with purified protein and cultured cells have revealed that caspases are susceptible to oxidative inactivation (14, 15, 16, 17). This study has shown that oxidation can occur at low concentrations of HOSCN, even in the presence of cellular reductants, making oxidative modification of caspases a distinct possibility at sites of inflammation.

The heme peroxidases lactoperoxidase, myeloperoxidase, eosinophil peroxidase, and peroxidasin use hydrogen peroxide to oxidize thiocyanate to HOSCN (19). LPO is constitutively active in epithelial lining fluid, while infiltration of neutrophils and eosinophils to a site of inflammation will dramatically increase levels of their respective peroxidases. At physiological pH, HOSCN is predominantly present as the OSCN^−^ anion, but HOSCN is the major oxidizing species. In contrast to hypohalous acids such as HOCl, HOSCN is more selective in its reactivity and specifically targets cysteine and selenocysteine residues in biological systems (38). This selectivity increases the lifetime and diffusion distance of HOSCN in biological systems, and as such, it will be a more effective caspase inhibitor than other reactive oxidants that are more indiscriminate in their reactivity profiles. It has been suggested that increased thiocyanate levels might protect against tissue damage by favoring HOSCN over HOCl production (38). However, our data suggest that the propensity for HOSCN to inhibit caspases and promote necroptosis might have the opposite effect by exacerbating inflammation.

Our results also indicate that HOSCN is considerably more effective at inactivating caspase-8 than caspase-3. More investigation is needed to determine what features of the caspase-8 active site make Cys360 so susceptible to oxidative inactivation, but we identified a disulfide between Cys360 and Cys409 as the major product of caspase-8 oxidation by HOSCN. In studies with the recombinant protein, where autocatalytic cleavage of pro-caspase-8 results in generation of p18 and p10 subunits, the modification is an intermolecular disulfide that is detectable by western blotting following non-reducing PAGE. In cells, modification with HOSCN was also apparent from the dramatic loss of the p18 and p10 subunits. In contrast to recombinant protein, a reversible dimer band was not detectable at the expected position on the gel, indicating more extensive modifications. Dot blotting confirmed that the antibodies were able to recognize the modified caspase-8, and our gel filtration and sedimentation experiments suggest that the protein formed higher molecular weight complexes. Caspase-8 binds to Fas-associated death domain (FADD). The FADD complex forms large assemblies of helical filaments whose elongation is controlled by the binding of other regulatory partners (36). Further investigation is required to determine the fate of oxidized caspase-8 in this more complex environment. Such knowledge will be invaluable for monitoring caspase-8 modification in tissues, especially in the digestive and respiratory tracts. Not only do these tissues have high levels of thiocyanate and increased heme peroxidase activity from infiltrating neutrophils, but necroptotic cell death is also implicated in inflammatory bowel disease (39) and pulmonary disease (40). There is also considerable interest in defining the cellular componentry that predisposes various tissues to undergo necroptosis, while others are less susceptible. Our study raises the prospect that an oxidizing environment, as promoted by the innate immune system, can condition cells to undergo necroptosis by inhibition of caspase-8 activity.

## Experimental procedures

### Reagents

Smac mimetic LCL161, recombinant (active) human caspase-3 (ALX-201-059), substrates Ac-DEVD-MCA (caspase-3), and Ac-IETD-MCA (caspase-8), and inhibitor (zVAD-fmk) were from Sapphire Bioscience (Sapphire Bioscience Pty Ltd). Recombinant (active) caspase-8 was expressed and purified from *E. coli* using established procedures (41). Anti-phospho-MLKL (ab196436) antibody was from Abcam, and anti-caspase-8 (1C12, #9746) and anti-cleaved caspase-8 (small subunit, #9748) were from Cell Signaling Technology. Nitrocellulose membrane and ECL Select Western Blotting Detection Reagent were from GE Healthcare. Secondary antibodies were from DAKO (Agilent). All cell culture media: Dulbecco modified Eagle medium, Roswell Park Memorial Institute 1640 (RPMI) medium, Medium 199 (M199), fetal bovine serum (FBS), penicillin/streptomycin (Pen/Strep) and TrypLE Express, and human TNF-α were from Thermo Fisher Scientific. Precast Mini-Protean gels (including TGX Stain-Free) and prestained protein markers were from Bio-Rad Laboratories Inc. All other reagents, including anti-MLKL (MABC604) and anti-β-actin (A5316) antibodies, were from Merck.

### Generation of HOSCN

HOSCN was generated enzymatically with bovine LPO as described previously (17). Briefly, LPO (100 μg) was added to sodium thiocyanate (7.5 mM) in 10 mM potassium phosphate buffer (pH 6.6), and 10 μl/ml H_2_O_2_ (from 75 mM stock) was added, in four separate aliquots, at 1 min intervals. LPO was then removed using centrifugation filters (10 kDa) at 13,000 rpm for 10 min at 4 °C. The yield of HOSCN was determined by measuring the change in absorbance of 5-thio-2-nitrobenzoic acid (TNB) at 412 nm and using the molar extinction coefficient for TNB (14,100 M^−1^ cm^−1^) then adjusting for the 1:2 stoichiometry of the reaction (HOSCN:TNB). To control for the potential effects of the decomposition products of HOSCN, the oxidant was left at room temperature for 5 h prior to use.

### Cell culture

Mouse dermal fibroblasts were generated from the tails of wildtype and *Mlkl*^*−/−*^ C57BL/6J mice and immortalized by SV40 large T antigen, as previously reported (20). Cells were maintained in Dulbecco modified Eagle medium with 10% FBS and 1% Pen/Strep and seeded at 75,000 to 100,000/cm^2^ in 24-well culture dishes 24 h prior to experimental treatments. For experiments, the media was switched to M199 with 10% FBS and 1% Pen/Strep as HOSCN is less reactive with constituents of this medium.

Apoptosis was induced with 5 μM of the Smac mimetic LCL161 (S) and 50 ng/ml of TNF-α (TS). Necroptosis was induced with 5 μM LCL161, 20 μM of the synthetic caspase inhibitor zVAD-fmk, and 50 ng/ml TNF-α (TSZ). The cells were preincubated with LCL161 and zVAD-fmk for 1 h prior to the addition of TNF-α. The point at which TNF-α was added was considered time zero for the timing of induction of apoptosis and necroptosis. When used, the necroptosis inhibitor Necrostatin 1 (Nec1) was also added 1 h before TNF-α. Concentrated Nec1, LCL161, and zVAD-fmk stocks were made in dimethyl sulfoxide (DMSO) with 0.15% the maximum amount of DMSO added to cells. Where appropriate, DMSO was used as “control.” HOSCN was generated immediately before use and added 10 min before TNF-α.

Jurkat T lymphoma cells (wildtype, TIB-152 clone E6-1 from ATCC, and caspase 8^−/−^ from Sanford Burnham Prebys Medical Discovery Institute) were cultured in RPMI with 10% FBS and 1% Pen/Strep, at 10^6^ cells/ml. Wildtype cells were exposed to TS for 2 h before the addition of HOSCN (TSOx) for 10 min, and caspase-8^−/−^ cells were used as a negative control for dot blotting.

### Recombinant protein production

Human caspase-8 (residues 217–479) was purchased from GenScript in a pET-30a vector harboring kanamycin resistance and containing a C-terminal hexahistidine tag and TEV protease cleavage site. The plasmid was transformed into BL21-DE3 pLysS *E. coli* cells and expressed at 20 °C for 4 h in 2xYT media. Cells were harvested and resuspended in lysis buffer (2 mM 2-mercaptoethanol, 20 mM imidazole, 50 mM Tris, 100 mM NaCl pH 8.0) and lysed by sonication. Soluble protein was separated by centrifugation and applied to Ni-NTA agarose beads (Qiagen). The elution from the Ni-NTA column was diluted 1:10 in low salt buffer (20 mM Tris pH 8.0) before application to a HiTrap Q FF anion exchange column (Cytiva). The protein was eluted using a continuous gradient from 10 mM to 1 M NaCl over 50 ml. The caspase-8 fraction was confirmed by performing an activity assay and then applied to a Hiload 16/600 Superdex 75 pg gel filtration column (using 20 mM Tris, 100 mM NaCl pH 8.0 and operating at 4 °C). After digestion overnight at 4 °C with TEV protease, the pure protein was obtained after application to a Ni-NTA column to remove the cleavage tag and hexahistidine-tagged TEV protease. The protein was desalted into 50 mM phosphate pH 7.4 using a Hiprep 26/10 desalting column (Cytiva) and stored at −80 °C.

### Caspase activity assays

Recombinant caspase-8 (25 μM), in buffer containing Tris (50 mM, pH 8.0), NaCl (100 mM), and imidazole (150 mM), was reduced with DTT (100 μM) prior to assay. The stock enzyme was diluted 1 in 100 for *in vitro* assays, therefore DTT was present at 1 μM in those assays. Recombinant caspase-3 from Sapphire Bioscience was reconstituted (0.06 μg/μl) in PBS containing 15% glycerol and stored at −80 °C. Prior to use, the enzyme was diluted in activity assay buffer (100 mM Hepes, pH 7.25 with 10% sucrose) and reduced with DTT (10 mM) and then Sepharose gel mini-spin columns were used to remove the DTT. Caspase activity was measured by fluorescent microplate assay using a Varioskan Flash multimode reader (ThermoFisher). One unit, for caspase-3, and 0.25 μM for caspase-8, was used per reaction; this was added directly to the assay plate (black, flat-bottomed 96-well format) and assay buffer, containing the appropriate substrate (50 μM), was added to a final volume of 90 μl. The final concentration of DTT was estimated to be 10 μM for caspase-3 and 1 μM for caspase-8. The reaction was monitored for 3 min (Ex 390 nm, Em 460 nm), then HOSCN was added (10 μl/reaction) and monitoring resumed for 3 min. Finally, DTT was added (10 mM for caspase-3, and 100 μM or 500 μM for caspase-8), and the reaction monitored for a further 3 min. The reaction rate (slope/sec) was calculated over 2 min at each stage of the assay and the rate expressed either as a percentage of the “0” (phosphate buffer, pH 6.6) control for both “+HOSCN” and “+DTT,” or as a percentage of the initial rate prior to addition of HOSCN.

Cellular caspase-3 activity was measured in MDF using the microplate assay; cells, including those having detached, were harvested with trypsin, washed once in PBS, then resuspended in 10 μl PBS for addition to the assay plate. Assay buffer (100 μl containing 100 mM Hepes, pH 7.25, 10% sucrose, 0.4% CHAPS and 0.1% Igepal CA-630) containing the enzyme substrate was dispensed into each well and the reaction monitored for 30 min. Enzyme activity (slope/min) was expressed as a percentage of the untreated control.

### Flow cytometry and imaging

Cells (MDF) were harvested, resuspended in 200 μl PBS with propidium iodide (25 μg), and cell death was measured using a Cytomics FC 500 flow cytometry system (Beckman Coulter). Ten thousand cells were analyzed per sample. Phase-contrast images were captured using the Olympus DP21 camera and the 10× magnification objective.

### Immunoblotting

MDF were harvested on ice at 30, 60, 90, or 120 min. Medium was collected, cells were washed twice with ice-cold PBS (also collected), and all detached cells were pelleted by centrifugation (4000 rpm) at 4 °C for 2 min. Lysis buffer (50 mM Tris-HCl, pH 7.5, 250 mM NaCl, 2 mM EDTA, 1% Igepal CA-630, 2 mM phenylmethylsulfonyl fluoride, 200 μM sodium vanadate, and 30 mM tetra-sodium pyrophosphate with protease and phosphatase inhibitors) was spread across the well surface and left on ice for 5 min. Lysed cells were then scraped and transferred to 1.7 ml tubes along with the pelleted, detached cells. These were kept on ice for the duration of the experiment, with occasional vortex mixing, then clarified by centrifugation (14,000 rpm) at 4 °C for 20 min and stored at −20 °C. Proteins (20 μg/sample) were separated by reducing SDS-PAGE (12%) and transferred to nitrocellulose membrane. Membranes were blocked for 1 h with 5% bovine serum albumin (BSA) in Tris-buffered saline (20 mM Tris, pH 7.6, 140 mM NaCl) with 0.05% Tween 20 (TBST) and incubated overnight with anti-phospho-MLKL antibody (ab196436, 1/1000) primary antibody in 5% BSA/TBST at 4 °C. The secondary antibody was goat anti-rabbit peroxidase (1/6000) in 3% BSA. Membranes were stripped of antibodies by incubation with Tris buffer (pH 6.8) containing SDS (2%) and 2-mercaptoethanol (100 mM) for 30 min at 50 °C. After washing and blocking for 1 h with 5% nonfat milk in TBST, membranes were incubated overnight with anti-MLKL antibody (MABC604, 1/3000 in 3% milk/TBST) and then for 1 h with rabbit anti-rat peroxidase secondary (1/6000). Membranes were subsequently washed and incubated for 4 h with anti-β-actin primary, followed by 1 h with goat-anti-mouse peroxidase secondary antibodies (10,000 5% milk/TBST).

Jurkat cells were harvested, lysed (20 mM Hepes, pH 7.5, 100 mM NaCl, 1% Igepal CA-630, 2 mM phenylmethylsulfonyl fluoride, 200 μM sodium vanadate with protease inhibitors) and separated by 20% SDS-PAGE, before immunoblotting with antibodies against caspase-8 to detect the proenzyme (55/57 kDa) and cleaved products at 41/43 kDa and 18 kDa, but not the small subunit. The same samples were also dot blotted to confirm that HOSCN treatment did not abrogate recognition by the antibodies: 2 μl of each sample was dotted onto dry nitrocellulose membrane and allowed to dry for 1 h. The membrane was then blocked and immunoblotted as for the western blot.

After exposure to HOSCN, recombinant caspase-8 was separated by 20% SDS-PAGE and immunoblotted separately for the large (p18) and small subunits (p10) to detect the formation, and resolution by DTT, of an intersubunit disulfide.

Gels were stained with Coomassie Brilliant Blue R250 to visualize recombinant proteins, and immunoblotted proteins were detected using ECL Select Western Blotting Detection Reagent with the Alliance Q9 Imaging system and quantified using UVBand software (UVTEC). Total proteins were visualized using TGX Stain-Free precast gels with UV imaging of trihalo-labeled tryptophan residues. MLKL phosphorylation levels were adjusted for the amount of total MLKL protein and expressed as a percentage of the relevant control.

### Treatment of caspase-8 for mass spectrometry analysis

Reduced caspase-8 (1.25 μM) in assay buffer was treated with 0 or 50 μM HOSCN for 10 min at room temperature. Where appropriate, GSH (100 μM) or dimedone (20 mM) was added to the samples and mixed before the addition of HOSCN.

### Whole protein LC/MS analysis

Samples were incubated with or without DTT (2.5 mM) for 30 min at room temperature. Whole protein samples were analyzed using a Velos Pro ion trap mass spectrometer, coupled to a Dionex Ulti-Mate 3000 HPLC system, with a 50 μl injection loop (Thermo Fisher Scientific). Samples were stored on the autosampler tray at 5 °C. An Accucore-15-C4 HPLC column (50 mm × 2.1 mm, 2.6 μm; Thermo Fisher Scientific) was used for chromatographic separation using 100% water (0.1% formic acid) as solvent A and 100% acetonitrile (0.1% formic acid) as solvent B. The column temperature was set to 60 °C. The column was equilibrated with 90% solvent A and 10% solvent B for 2.5 min and then a linear gradient was run for 2.1 min to 20% solvent A and 80% solvent B to elute the proteins. The column was then flushed with 20% solvent A and 80% solvent B for 2.5 min and reequilibrated at initial conditions for 2.5 min. A flow rate of 0.4 ml/min was used, and 2 μg of protein was injected for each sample. Nitrogen was used as sheath gas. The temperature of the heated capillary was 275 °C. Mass spectral data were acquired from 3 to 9 min of each chromatographic separation, scanning between m/z 410 and 2000 in positive-ion mode at a normal scan rate. Spectra were averaged over the full length of each protein peak using Thermo Xcalibur Qual Browser 2.2 SP1.48 (Thermo Fisher Scientific) and deconvoluted to yield the molecular masses using ProMass for Xcalibur (Version 2.8 rev. 5; Novatia LLC).

### Tryptic digest LC/MS analysis

Samples were incubated with or without DTT (2.5 mM) for 30 min at room temperature, followed by alkylation with 50 mM iodoacetamide for 30 min in the absence of light. Trypsin was added to samples at a 20:1 (substrate:trypsin) weight ratio and incubated at 37 °C overnight. Digested samples were analyzed using a Velos Pro ion trap mass spectrometer coupled to a Dionex Ulti-Mate 3000 HPLC system with a 50 μl injection loop (Thermo Fisher Scientific). Samples were stored on the autosampler tray at 5 °C. A Jupiter 4-μm Proteo 90 Å column (150 × 2 mm, Phenomenex) was used for chromatographic separation using 100% water (0.1% formic acid) as solvent A and 100% acetonitrile (0.1% formic acid) as solvent B. The column temperature was set to 40 °C. The column was equilibrated with 95% solvent A and 5% solvent B for 5 min, and then a linear gradient was run for 45 min to 5% solvent A and 95% solvent B to achieve separation. The column was then flushed with 5% solvent A and 95% solvent B for 5 min and re-equilibrated at initial conditions for 5 min. A flow rate of 0.2 ml/min was used, and 2 μg of digested protein was injected for each sample. Nitrogen was used as sheath gas. The temperature of the heated capillary was 275 °C. Data were analyzed using Thermo Xcalibur Qual Browser 2.2 SP1.48 (Thermo Fisher Scientific).

**Supplementary Figure 1:**
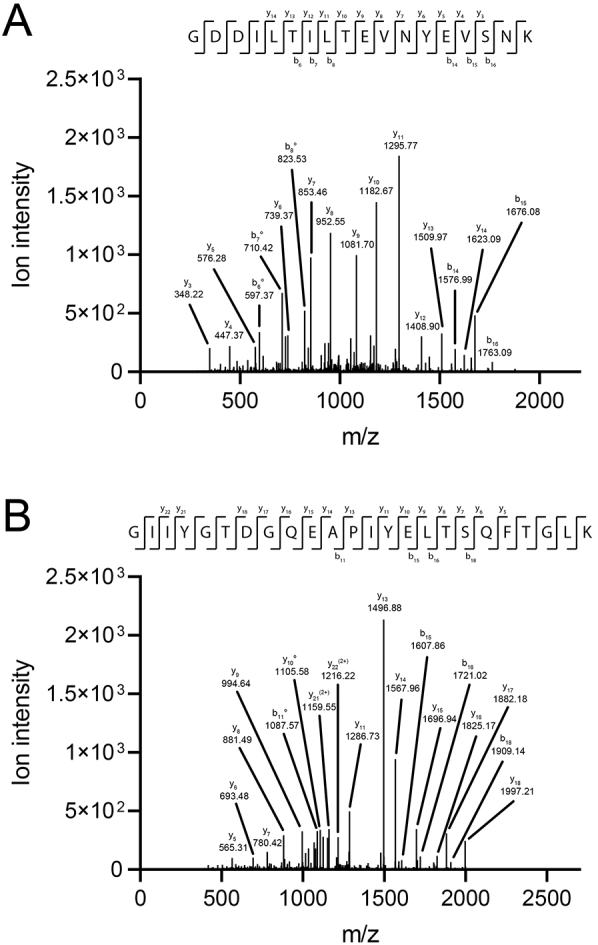
**Fragmentation patterns of control caspase-8 peptides**. *A*, CID-MS/MS spectrum for the [M + 2H]^2+^ ion of the GDDILTILTEVNYEVSNK peptide (1012.02 m/z). *B*, CID-MS/MS spectrum for the [M + 2H]^2+^ ion of the GIIYGTDGQEAPIYELTSQFTGLK peptide (1301.16 m/z). The loss of water from a fragment ion is denoted by ‘°’ where y_x_° is y_x_-H_2_O.

**Supplementary Figure 2:**
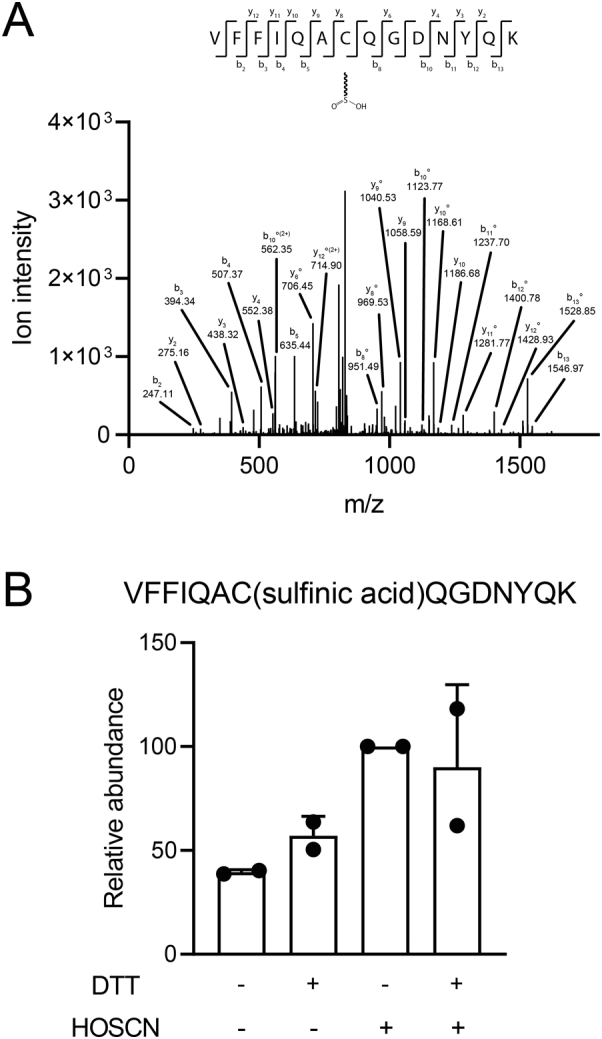
**Incubation of caspase-8 with HOSCN results in the formation of sulfinic acid**. Recombinant caspase-8 was untreated or treated with HOSCN (50 μM). Samples were split and half of each exposed to DTT (2.5 mM), then alkylated, and digested with trypsin, before analysis by LC-MS/MS. *A*, CID-MS/MS spectrum for the [M + 2H]^2+^ ion of the VFFIQAC_360_(sulfinic acid)QGDNYQK peptide (846.89 m/z). The loss of water from a fragment ion is denoted by ‘°’ where y_x_° is y_x_-H_2_O. *B*, the relative peak area for VFFIQAC_360_(sulfinic acid)QGDNYQK was determined for each treatment. Data are from two separate experiments. HOSCN, hypothiocyanous acid.

**Supplementary Figure 3:**
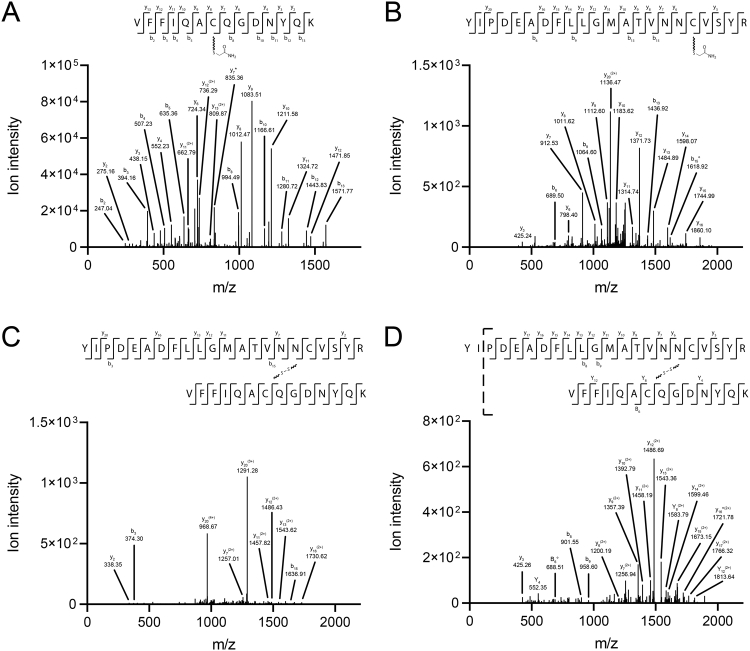
**Fragmentation patterns of VFFIQAC**_**360**_**QGDNYQK and YIPDEADFLLGMATVNNC**_**409**_**VSYR peptides**. *A*, CID-MS/MS spectrum for the [M + 2H]^2+^ ion of the VFFIQAC_360_(CAM)QGDNYQK peptide (859.40 m/z). *B*, CID-MS/MS spectrum for the [M + 2H]^2+^ ion of the YIPDEADFLLGMATVNNC_409_(CAM)VSYR peptide (1274.60 m/z). *C*, CID-MS/MS spectrum for the [M + 4H]^4+^ ion of the VFFIQAC_360_QGDNYQK-YIPDEADFLLGMATVNNC_409_VSYR disulfide peptide (1037.99 m/z). *D*, CID-MS/MS/MS spectrum for the y_20_^3+^ ion (1291.60 m/z) that results from fragmentation of the quadruply charged VFFIQAC_360_QGDNYQK-YIPDEADFLLGMATVNNC_409_VSYR disulfide peptide ion (1037.99 m/z). CAM: carbamidomethylation resulting from treatment with iodoacetamide. The loss of water from a fragment ion is denoted by ‘°’ where y_x_° is y_x_-H_2_O. The loss of ammonia from a fragment ion is denoted by ‘∗’ where y_x_∗ is y_x_-NH_3._

**Supplementary Figure 4:**
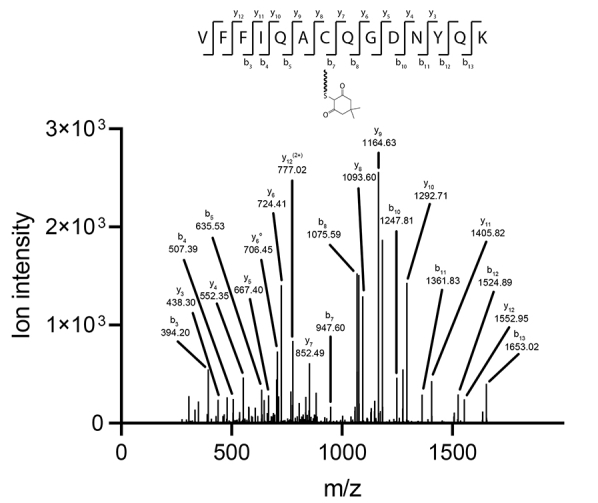
**Incubation of caspase-8 with HOSCN results in the formation of sulfenic acid.** Recombinant caspase-8 was treated with HOSCN (50 μM) in the presence of dimedone (20 mM), then alkylated and digested with trypsin, before analysis by LC-MS/MS. Representative CID-MS/MS spectrum for the [M + 2H]^2+^ ion of the VFFIQAC_360_(dimedone)QGDNYQK peptide (899.93 m/z). The loss of water from a fragment ion is denoted by ‘°’ where y_x_° is y_x_-H_2_O. HOSCN, hypothiocyanous acid.

**Supplementary Figure 5:**
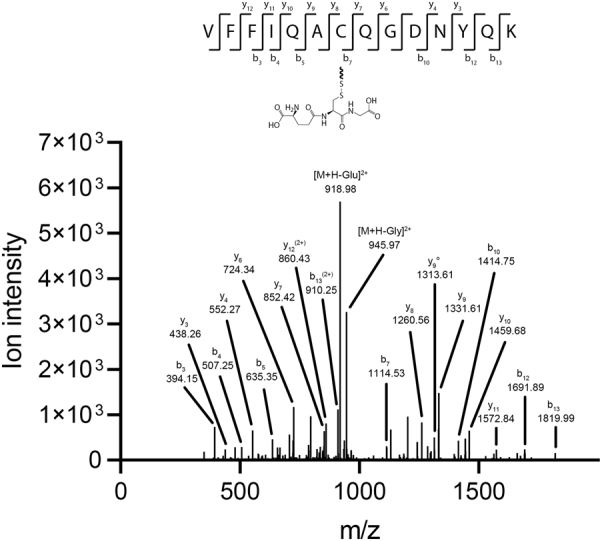
**Caspase-8 forms a mixed disulfide with GSH in the presence of HOSCN.** Recombinant caspase-8 was treated with HOSCN (50 μM) in the presence of GSH (100 μM), then alkylated and digested with trypsin, before analysis by LC-MS/MS. Representative CID-MS/MS spectrum for the [M + 2H]^2+^ ion of the VFFIQAC_360_(GSH)QGDNYQK peptide (983.43 m/z). The loss of water from a fragment ion is denoted by ‘°’ where y_x_° is y_x_-H_2_O. HOSCN, hypothiocyanous acid; GSH, glutathione.

**Supplementary Figure 6:**
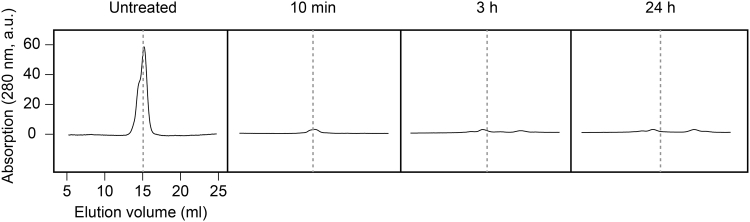
**Gel filtration of oxidized recombinant caspase-8.** Recombinant caspase-8 was exposed to HOSCN (100 μM) for 10 min, 3 h or 24 h before analysis by gel filtration on a Superdex 200 increase 10/300 GL column. HOSCN, hypothiocyanous acid.

The m/z values of peptides of interest were predicted, and collision-induced dissociation-MS/MS spectra in positive-ion mode were acquired for each of them. The collision energy was set at 40. Peptide fragments were manually assigned based on Roepstorff-Fohlman nomenclature (42). Each peptide species was quantified by postacquisition filtering the MS/MS spectra obtained for a chosen abundant and characteristic fragment ion (listed in Table S1) and then measuring the area under the curve of the resulting peak (peak algorithm: Genesis, peak smoothing: Gaussian 7 points). Peptide peak areas were normalized by dividing by the sum of the peak areas of two peptides (GDDILTILTEVNYEVSNK and GIIYGTDGQEAPIYELTSQFTGLK, Fig. S1) that were unaffected by treatment.

### Statistical analysis

Data are expressed as means and SD of the indicated number of independent experiments. Details of the statistical analyses used are given in the figure legends, and differences were considered significant when the *p* value was less than 0.05. All analyses were performed using GraphPad Prism, version 9.2.0 (GraphPad Software).

## Data availability

All data are contained within the manuscript.

## Supporting information

This article contains supporting information.

## Conflict of interest

J. M. M. contributes to the development of necroptosis inhibitors in collaboration with Anaxis Pharma Pty Ltd. G.S.S. consults for Genentech Inc. All other authors declare they have no conflicts of interest with the contents of this article.
