# Supplementary material for: Oxidation of caspase-8 by hypothiocyanous acid enables TNF-mediated necroptosis
Source: J Biol Chem. 2023 May 6;299(6):104792. doi: 10.1016/j.jbc.2023.104792 (PMC10267563; doi:10.1016/j.jbc.2023.104792)
Supplement: Supplementary Table 1 [file mmc1.docx]

**Supplementary Table 1. The m/z values for the parent ions and the daughter fragment ions that were used to quantify each peptide in LC-MS/MS experiments.** CAM: carbamidomethylation resulting from treatment with iodoacetamide.

| **Peptide (charge state)** | **Parent ion** | **Fragment ion** |
| --- | --- | --- |
| VFFIQAC_360_(sulfinic acid)QGDNYQK (+2) | 846.89 m/z | y_10_-H_2_O: 1168.47 m/z |
| VFFIQAC_360_(CAM)QGDNYQK (+2) | 859.40 m/z | y_9_: 1083.45 m/z |
| YIPDEADFLLGMATVNNC_409_(CAM)VSYR (+2) | 1274.60 m/z | y_12_: 1371.61 m/z |
| VFFIQAC_360_QGDNYQK-YIPDEADFLLGMATVNNC_409_VSYR disulfide (+4) | 1037.99 m/z | y_20_^3+^: 1291.60 m/z |
| VFFIQAC_360_(dimedone)QGDNYQK (+2) | 899.93 m/z | y_9_: 1164.50 m/z |
| GDDILTILTEVNYEVSNK (+2) | 1012.02 m/z | y_11_: 1295.65 m/z |
| GIIYGTDGQEAPIYELTSQFTGLK (+2) | 1301.16 m/z | y_13_: 1496.80 m/z |
