# Supplementary material for: Oxidation of caspase-8 by hypothiocyanous acid enables TNF-mediated necroptosis
Source: J Biol Chem. 2023 May 6;299(6):104792. doi: 10.1016/j.jbc.2023.104792 (PMC10267563; doi:10.1016/j.jbc.2023.104792)
Supplement: Supplementary Table 2 [file mmc2.docx]

**Supplementary Table 2. The expected molecular weights of caspase-8 species.**

| **Species** | **Average mass (Da)** |
| --- | --- |
| p10 | 10889.3 |
| p10 + sulfinic acid | 10921.3 |
| p10 + GSH | 11194.6 |
| p18 | 20065.5 |
| p18 + sulfinic acid | 20097.5 |
| p18 + GSH | 20370.8 |
| p10-p18 disulfide | 30952.8 |
